# Altered oscillatory activity pattern during reconfiguration processes of perception–action representations in adolescents with AD(H)D

**DOI:** 10.1016/j.nicl.2025.103905

**Published:** 2025-11-08

**Authors:** Katharina Graf, Roula Jamous, Annet Bluschke, Christian Beste

**Affiliations:** a Cognitive Neurophysiology, Department of Child and Adolescent Psychiatry, Faculty of Medicine, TU Dresden, Germany; b German Center for Child and Adolescent Health (DZKJ), partner site Leipzig/Dresden, Dresden, Germany

**Keywords:** ADHD, EEG, Theta band activity, Alpha band activity, Perception–action integration, Executive dysfunction, Adolescents

## Abstract

Deficiencies in cognitive control have been commonly associated with AD(H)D. Yet, perception–action integration, one of the fundamental components of cognition and central to goal-directed behavior, and its underlying neurophysiological mechanisms have received little attention in AD(H)D research. An investigation of these mechanisms might help to aim for a more comprehensive understanding of neuropsychological models of the disorder, and by that help to provide insights into how cognitive control and executive functioning deficits may arise in AD(H)D. In the current study, we therefore examined both behavioral performance and oscillatory activity of perception–action integration processes in children and adolescents (9–17 years of age) with AD(H)D (n = 30) compared to a neurotypical control group (n = 38) using a simplified version of an established event-file paradigm. Overall, study results suggest that individuals with AD(H)D experience greater difficulties in the reconfiguration process of updating earlier established perception–action representations. Behaviorally, this was indicated not only by the poorer general performance of the AD(H)D group but especially in binding incompatible trials, namely trials requiring reconfiguration processes. Neurophysiological data indicate that behavioral deficits in AD(H)D likely develop due to a reduced ability to modulate alpha-band activity when updating existing perception–action representations, with no significant alpha-band modulated differences across task demands, rather than due to impairments in theta band modulation. By this, the study highlights the importance of modulating both theta and alpha band activity for the successful management of perception–action representations, and further supports the assumption that AD(H)D might reflect a disorder of deficient alpha band modulation.

## Highlights

• AD(H)D adolescents struggle more to reconfigure perception–action links.
• Poor behavioral performance was specifically evident in binding incompatible trials.
• Reduced alpha band modulation when updating perception–action links in AD(H)D.

## 1 Introduction

Deficiencies in cognitive control and executive functioning have been commonly associated with and reported to play a significant role in attention-deficit(−hyperactivity) disorder (AD(H)D), one of the most predominant neuropsychiatric disorders of childhood and adolescence (Ahmadi et al., 2014, Association, 2013, Barkley, 1997, Castellanos and Tannock, 2002, Randall et al., 2009). Intact cognitive control abilities are essential for achieving any kind of goal-directed actions (Diamond, 2013). Thus, impairments in these functions adversely affect daily functioning and are linked to symptom severity in individuals with AD(H)D (Biederman et al., 2004, Loo et al., 2007, Willcutt et al., 2005). Yet, perception–action integration, as one of the fundamental components of cognition and central to goal-directed behavior (Hommel et al., 2001), has not received much attention in AD(H)D research. This is also, and especially, the case for the underlying neurophysiological mechanisms of perception–action representations. An investigation of these mechanisms in adolescents with AD(H)D may help to aim for a better, more comprehensive understanding of neuropsychological models of the disorder. It may further help to adapt more targeted future treatment approaches and training programs aimed at enhancing executive functioning, ultimately improving the daily life abilities of affected individuals.

The Binding and Retrieval of Action Coding (BRAC; Frings et al., 2020) framework, which builds upon the Theory of Event Coding (TEC; Hommel, 1998, Hommel et al., 2001), offers a well-recognized cognitive science-driven framework to analyze perception–action integration processes. According to these frameworks, the characteristics of stimuli, responses, and their outcomes are retained as internal representations in an episodic memory entry, i.e., perception–action representations. These entire mental representations, also referred to as event-file bindings, are retrieved as soon as any of the components (stimulus, response, associated actions) of the event-file network is repeated. Depending on the demands of a given situation, retrieving an earlier established stimulus–response association can either facilitate or impede the following actions. If stimulus–response associations do not align with previously established ones, event-file bindings need to be reconfigured by rejecting existing mental representations and updating these with new stimulus–response associations. Importantly, BRAC expands upon the theoretical framework of TEC by treating feature retrieval and binding as distinct processes (Frings et al., 2020). More recently, these conceptions suggest that different neural oscillatory activities are central to the brain’s information processing, and mechanisms were proposed for how concurrent activity across frequency bands complement each other to enable efficient perception–action integration processes as part of fronto-parietal circuits (Beste et al., 2023).

Oscillatory activity specifically in the theta (∼4-7 Hz) and alpha (∼8-12 Hz) frequency bands was suggested to play a crucial role in managing integrated perception–action representations (Beste et al., 2023, Graf et al., 2024, Prochnow et al., 2022, Prochnow et al., 2025, Rawish et al., 2024, Wendiggensen et al., 2023). While theta band activity (TBA) was proposed to represent the continuous integration (binding) and retrieval processes of recently created perception–action representations into an episodic memory trace, evidence accumulates that alpha band activity (ABA) may exert bottom-up attentional processes as well as top-down control over these theta band-related processes (Beste et al., 2023). Furthermore, research indicated that TBA is crucial for error and conflict detection since it is enhanced and associated with better performance on a variety of cognitive control tasks, including conflict monitoring (Cavanagh et al., 2009, Eschmann et al., 2018, Trujillo and Allen, 2007) and working memory updating (Itthipuripat et al., 2013, Jacobs et al., 2006, Jensen and Tesche, 2002, Klimesch et al., 2006). In contrast, ABA was shown to facilitate the processing of relevant information by implementing control in a top-down manner to actively suppress distracting or task-irrelevant cognitive representations in working memory (Bonnefond and Jensen, 2012, Foxe and Snyder, 2011, Janssens et al., 2018, Klimesch et al., 2007). ABA was further suggested to be modulated and increased by working memory and attentional load as a means to maintain preexisting mental representations from potential interference or competition prompted by novel information (Janssens et al., 2018, Jensen et al., 2002, Lenartowicz et al., 2018). Understanding how TBA and ABA contribute to the management of perception–action representations is thus crucial in understanding how potential deficits in adolescents with AD(H)D may develop.

Several aspects give rise to the idea that adolescents with AD(H)D may be characterized by altered perception–action representations. Particularly, extensive brain oscillatory changes have been proposed to be related to AD(H)D (McLoughlin et al., 2022, Michelini et al., 2022). TBA-related executive functioning deficits in AD(H)D were reported, such as demonstrating weaker midfrontal TBA in tasks involving high levels of cognitive conflict (Johnson et al., 2008, Lenartowicz et al., 2014, McLoughlin et al., 2022, McLoughlin et al., 2014). The role of less efficient regulation of ABA in AD(H)D has recently gained more attention, and evidence emerged that AD(H)D might reflect a disorder of deficient alpha band modulation (Gholamipourbarogh et al., 2025, Graf et al., 2024, Ippolito et al., 2022, Lenartowicz et al., 2014, Lenartowicz et al., 2019, Michelini et al., 2022). Notably, a recent study indicated that adolescents with AD(H)D show a weakened ability to efficiently reconfigure already established perception–action representations in the working memory during a response inhibition task, which was possibly linked to inefficient and delayed regulation of the alpha band (Graf et al., 2024). Interestingly, these impairments did not seem to result from a generally compromised activation of critical brain areas or inadequate modulation of TBA. Furthermore, the integration of perceptions and their associated actions is closely linked to the involvement of prefrontal regions as well as dopaminergic neurotransmission (Colzato et al., 2012, Colzato et al., 2013, Colzato and Hommel, 2008, Elsner et al., 2002, Kühn et al., 2011, Melcher et al., 2014, Petruo et al., 2016, Zmigrod et al., 2014) – both of which have been reported to be altered in individuals with AD(H)D (Arnsten, 2009, Bush, 2011; Silk et al., 2008. Hence, accumulating evidence underscores the importance of investigating the neural underpinnings of perception–action integration processes during response selection to develop a more nuanced view of how executive functioning deficits arise in AD(H)D.

In the current study, we therefore examine both behavioral performance (percentage of correct responses, reaction time in milliseconds) and the underlying oscillatory activity – specifically the modulation of ABA and TBA − of perception–action integration processes in adolescents with AD(H)D compared to a neurotypical (NT) control group on an adjusted version of the stimulus–response (S-R) task (Colzato et al., 2006a, Colzato et al., 2006b; please see *Method* section for further details on the adjustments made). The task includes 1) conditions in which previously established perception–action representations remain intact and do not need to be reconfigured (i.e., binding compatible trials) and 2) conditions in which previously established perception–action representations need to be reconfigured (i.e., binding incompatible trials). Time-frequency analysis is applied to study the underlying modulations of oscillatory activity during perception–action integration processes. Source-localization methods (DICS beamforming analysis) are used to localize the involved brain areas related to differences in oscillatory modulation for the contrast between binding compatible compared to binding incompatible trials. In doing so, we aim to elucidate how potential time–frequency group differences are related to possible differences in activated brain regions based on the reconstructed sources from EEG sensor-level data.

Considering the literature discussed above, we hypothesize that adolescents with AD(H)D will show an abnormal management of perception–action integration processes compared to the NT control group. Behaviorally, this would be indicated by adolescents with AD(H)D performing not only worse overall, compared to the NT control group, but also showing a greater difference in binding compatible compared to binding incompatible trials. Hence, they are suggested to have greater difficulties when a previously established stimulus–response association needs to be reconfigured. On the neurophysiological level, we expect adolescents with AD(H)D to be less successful in modulating theta and alpha band activity, specifically in response to binding incompatible trials. Compared to NT controls, this would imply poorer management of cognitive monitoring processes and a poorer ability to update perception–action representations in the working memory. Further, inefficient or possibly delayed modulation in the alpha band may indicate a reduced ability to suppress task-irrelevant cognitive representations in working memory.

## 2 Materials and methods

### 2.1 Sample

Participants were recruited using an in-house database containing contact details and medical/demographic information (such as IQ values and psychiatric diagnoses) with consent from the (possible) participants and their legal guardians. AD(H)D patients in this in-house database were originally recruited by the outpatient clinic affiliated with our department. During a brief telephone interview, all (possible) participants and their legal guardians were asked about their interest in study participation and whether they met the inclusion/exclusion criteria of the current study.

A total of N = 95 adolescents (9–17 years) were included in the study, of whom N = 48 adolescents had a confirmed AD(H)D diagnosis according to the ICD-10 (AD(H)D group) and N = 47 adolescents had no prior reported psychiatric diagnosis and served as a neurotypical control group (NT group). Prior to data analysis, N = 18 participants of the AD(H)D group and N = 9 participants of the NT group had to be excluded for several reasons. Of the originally N = 48 recruited and conducted testing of the AD(H)D sample, N = 16 participants had to be excluded before data analysis because they performed below chance level (at least 50 % correct trials per condition needed) and/or were detected as extreme outliers of the group. Of the behaviorally included participants, two further participants had too poor EEG data quality to ensure reliable analysis, resulting in too few segments (<15) to analyze after manual preprocessing. The mean IQ of the final sample of N = 30 included AD(H)D participants was average (m = 105.57, SE = 2.085; 87–123; two participants had missing reported IQ values). Participants were only eligible to take part in the study if AD(H)D medication was used in the morning, and study participation took place in the late afternoon. This way, we hope to reduce the likelihood of medication effects on study performance (Morton & Stock, 2000). Within the NT control group, of initially N = 47 recruited participants, N = 6 participants had to be excluded due to performance below chance level (at least 50 % correct trials per condition needed) and/or were detected as extreme outliers of the group, and further N = 3 participants because their EEG data quality was too poor, resulting in too few segments (<15) to analyze after manual preprocessing to ensure reliable analyses. By that, the final sample size of NT controls resulted in N = 38. Please refer to Table 1 for more information on demographic variables and reported psychiatric diagnoses. The study was approved by the local ethics committee and carried out in accordance with the Declaration of Helsinki (1964).

**Table 1:** *Demographics of the Sample* (*N_NT_* = *38, N_ADHD_ = 30*).

Demographic variables	NT Group (m ± SE)	ADHD Group (m ± SE)	Significance test
Gender (Valid *N*)			*χ^2^*(1) = 12.13, *p* < 0.001***
Male	13	23	
Female	25	7	
Age	13.74 ± 0.29	13.47 ± 0.36	*U* = 626.00, *p =* 0.487
Reported psychiatric diagnoses (Valid *N*)			
ADHD	0	17	*…*
ADD	0	10	
Hyperkinetic conduct disorder	0	3	
Other reported psychiatric diagnoses (Valid *N*)			
Dyslexia	0	2	*…*
Developmental coordination disorder	0	2	
Moderate depressive episode	0	1	
Disruptive Mood Dysregulation Disorder	0	1	

Note. 0.01 ≤ p < 0.05: *; 0.001 ≤ p < 0.01: **; p < 0.001: ***.

Abbreviations: NT = neurotypical, AD(H)D = attention-deficit(−hyperactivity)-disorder, SE = standard error.

#### 2.1.1 Power considerations

The final sample size is similar to other research investigating perception–action representations in psychiatric groups (Graf et al., 2024, Kleimaker et al., 2020, Prochnow et al., 2025, Takacs et al., 2024), and it enables the detection of a minimum effect size of f = 0.25 (i.e., η_p_^2^ = 0.06) with a 95 % power for the interaction ‘group x reconfiguration’ in the current study methodology (mixed effects ANOVA with two groups and two conditions; Faul et al., 2007). Yet, the great number of exclusions on the behavioral level of the AD(H)D group should be considered while drawing conclusions from the study results.

### 2.2 Task

A modified version of the stimulus–response (S-R) task was used to investigate event-file binding in the current study (Colzato, Warrens, et al., 2006). Earlier studies already demonstrated that the “zero feature” and “full feature” overlap conditions within randomly presented alternation and repetition response trials are most critical for accurately analyzing event-file coding processes (Kleimaker et al., 2020, Takacs et al., 2020a, Takacs et al., 2020b). Moreover, to simplify the task for adolescents with AD(H)D and reduce levels of frustration and length of the experiment, further modifications on the task were done: The response window for R1 was slightly increased by 200 ms (from 500 ms to 700 ms), repetition of incorrect trials were reduced from three to two, the number of breaks were increased from 5 to 11 to have smaller blocks of trials, short versions of the instructions were included into the breaks, and the instruction text was simplified (the German words “liegt” und “steht” instead of “waagerecht” and “senkrecht”).

**Fig. 1:**
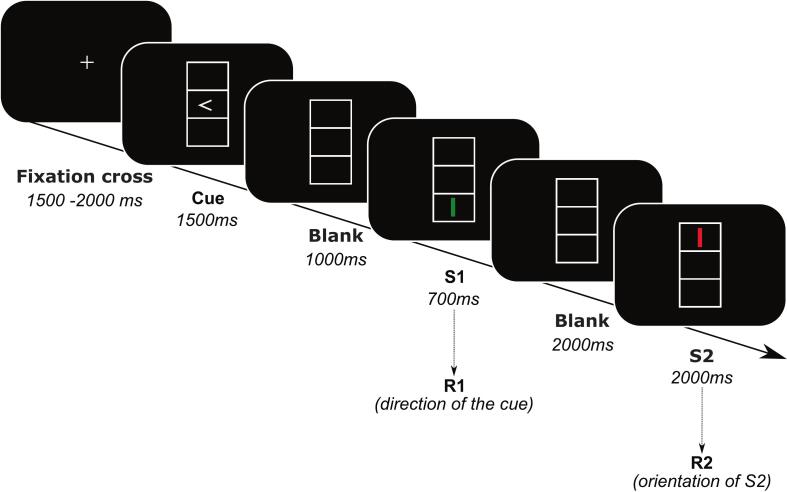
Schematic illustration of the stimulus–response (S-R) task. The figure displays the sequence of stimuli and responses during the adjusted stimulus–response (S-R) task (Colzato, Warrens, et al., 2006). Please refer to the Method section for detailed information regarding the adjustments made. This illustration was

Participants sat in front of a 24-inch computer screen located around 60 cm away from the participants. Following several practice trials, participants performed 192 trials broken down into twelve blocks of 16 trials each. The task structure was as follows: two stimuli were presented, one after the other, after a response cue appeared on the screen in one of three vertically aligned boxes (each measuring 2.4 x 0.9 cm; see Fig. 1). More specifically, the response cue – an arrowhead pointing either to the left or right – was shown in the middle box for 1500 ms after a fixation cross was shown on a blank screen for 1500 – 2000 ms between the trials. Following a 700 ms display of the first stimulus (S1), the second stimulus (S2) was displayed for 2000 ms, or until the participants had responded. An empty screen was displayed for 1000 ms between the response cue and S1 and for 2000 ms between S1 and S2. The stimuli varied in color (green, red) and orientation (vertical, horizontal), and they were either shown in the bottom or top box. As a result, task stimuli varied in three distinct aspects: color, orientation, and location. Per trial, two responses were demanded. Participants were told to wait for S1 to appear before responding to the direction of the response cue (R1). The trials were repeated up to two times for incorrect answers. Crucially, earlier studies demonstrated that an automated event-file binding is elicited by the evolving relationship/interaction between S1 and R1 (Hommel, 1998). Thereafter, the second response was required by reacting to the characteristics of the second stimulus (S2) itself at the moment of appearance of S2. The left control key needed to be pressed for a horizontal bar; the right control key for a vertically presented bar. S1 and S2 were either completely different from one another (“no overlap” condition, meaning that S1 and S2 did not share any stimulus characteristics) or were equivalent to one another (“full overlap” condition, meaning that S1 and S2 shared all stimulus characteristics). Consequently, during each trial, participants were required to repeat their response by pushing the same button twice or by alternating their responses by pushing a different button.

adopted from Graf et al. (2023).

### 2.3 EEG recording and preprocessing

During task performance, the EEG signal was recorded using 60 Ag/AgCl electrodes (Fpz as reference electrode, the ground electrode maintained at θ = 58, φ = 78, electrode impedance less than 5 kΩ). During the offline pre-processing of the data using BrainVision Analyzer 2.1, a band-pass filter of 0.5 – 40 Hz was used, and the sampling rate was adjusted from 500 Hz to 256 Hz. Periodic artifacts (e.g., pulse artifacts, horizontal and vertical eye movements) were corrected using an independent component analysis (ICA, infomax algorithm), while technical artifacts were eliminated after manual examination of the data. Subsequently, the data were stimulus-locked and segmented based on the stimulus onset of S2 (−2000 to 2000 ms).

In total, four distinct segments were created based on the level of feature overlap (full vs no overlap) and response type (repetition vs alternation), which were later broken down into two different conditions, namely binding incompatible trials (full overlap-alternation, no overlap-repetition) and binding compatible trials (full overlap-repetition, no overlap-alternation). This was done to compare trials in which an event-file binding needs to be reconfigured (i.e., binding incompatible) with trials in which an event-file binding does not need to be reconfigured (i.e., binding compatible) to each other on the neurophysiological level. EEG-epochs were only included in the segmentation for further analysis when responses R1 and R2 were accurate and when R1 appeared within 700 ms after the appearance of S1 in the task. Following the presentation of S2, R2 had to be given within 2000 ms.

After segmentation, the following rejection criteria were used in an automated artefact rejection procedure: Intervals with activity below 0.5 µV for 100 ms and amplitudes greater than ± 150 µV were eliminated and marked as poor intervals 200 ms before and 200 ms after the event, and segments containing these bad intervals were rejected for further analyses. Further, a baseline correction was applied (−200 – 0 ms before the onset of S2). Segments ranging from −200 to 1000 ms locked to S2 were used for all subsequent analyses.

### 2.4 Time-frequency decomposition

A time–frequency transformation using Morlet wavelets (Morlet parameter of 5) was carried out to compare oscillatory activity of the two conditions (binding incompatible, binding compatible), both within and between the two groups (NT controls, AD(H)D). The analysis was performed between 1 and 30 Hz in steps of 1 Hz. Non-parametric cluster-based permutation tests were conducted using FieldTrip (Maris & Oostenveld, 2007) to identify the electrodes and time windows with significant differences in alpha band activity (ABA; averaged over 8–––12 Hz) and theta band activity (TBA; averaged over 4 – 7 Hz) between the two conditions and the two groups. Two consecutive time points or two nearby EEG channels were required to define a cluster. Using the Monte Carlo approach, 1000 random draws were used to estimate the reference distribution of the permutation test. A cluster was considered significant if its matching p-value was less than 0.05. A global baseline normalization was applied for the time–frequency analyses to ensure that possible group differences do not result from differences in baseline oscillatory activity values (e.g., Lenartowicz et al., 2018). For that purpose, we first exported a baseline (−2000 – 1000 ms) locked to the first stimulus of the task (i.e., the cue) for each subject from BrainVision Analyzer 2.1 and imported this data into FieldTrip. Subsequently, the time–frequency analysis was calculated after normalizing the data for each group individually using the imported baseline (− 500 – 0 ms, averaged over time) integrated into the time–frequency analysis steps described earlier.

### 2.5 Beamforming

To reconstruct the alpha and theta source level activity from the EEG sensor-level data, we employed a multistep beamforming technique: At first, Dynamic Imaging of Coherent Sources (DICS) beamforming (Gross et al., 2001) was used to identify brain regions linked to activity modulations in the theta and alpha frequency bands to evaluate the source activity underlying these frequency bands for the contrast between binding incompatible and binding compatible trials in both groups. This was done to localize brain areas related to activity modulations in the different frequency bands. The DICS beamforming was calculated by computing common spatial filters within each group using the cross-frequency spectra of a Fast Fourier Transformation (FFT) on the averaged alpha (8–12 Hz) and averaged theta (4–7 Hz) frequency bands. The DICS beamformer used the forward model template from the FieldTrip toolbox, which is based on the standard Montreal Neurological Institute (MNI) space, to map the frequency band activity onto a grid with even spacing of 0.5 cm. The center-of-the-head bias was subsequently eliminated by computing the Neural Activity Index (NAI) for source reconstruction of the individual conditions. This was accomplished by dividing the source power estimates by the local noise per voxel estimates that corresponded to them (Van Veen et al., 1997). Using the Density-Based Spatial Clustering of Applications with Noise (DBSCAN) algorithm (Adelhöfer and Beste, 2020, Ester et al., 1996) as implemented in MATLAB (2020), all voxels revealed by the DICS beamformer were clustered individually for each frequency band of interest for the contrast trials. By manually examining the size and/or related AAL atlas labels, the DBSCAN analysis results were further limited to functionally significant clusters, and, as a result, big clusters spanning various neuroanatomical regions were separated into distinct clusters. According to the Automatic Anatomical Labeling (AAL) atlas (Tzourio-Mazoyer et al., 2002), all voxels within functional neuroanatomical regions and having source activity greater than two percent of the highest values were chosen for clustering. With a minimum cluster size of two voxels and an epsilon of once the edge length, DBSCAN was utilized to find nearby voxels. By subtracting the source power of binding incompatible trials from the source power of binding compatible trials, proportional to the summation of these two conditions, contrasts were calculated. The time windows for the beamforming analyses were based on the time windows for significant clusters as revealed by the time–frequency analysis for both frequency bands and groups. In case no significant clusters were revealed for a specific frequency band within a group, time windows were chosen based on the time windows of significant clusters of the other group. Since it was not feasible to perform individual brain scans, we used conventional adult brain templates for the study. As far as we know, no appropriate template exists that can accommodate children and adolescents of various ages while accounting for the brain’s ongoing development (Simmonds et al., 2014). Thus, this approach was used to get an indication of whether there may be a substantial difference in the origins of theta and alpha band activity between adolescents with and without AD(H)D. Yet, reconstructed sources and Brodmann areas mapped from these reconstructions should be interpreted with caution.

### 2.6 Statistical analysis

To keep behavioral and neurophysiological analyses aligned and comparable, two new conditions (binding compatible, binding incompatible) were created on both levels based on the original four distinct conditions developed from the level of feature overlap (full vs no) and response type (repetition vs alternation). Thus, on the behavioral level, binding compatible conditions were created by taking the mean value of the ‘full overlap-repetition’ and ‘no overlap-alternation’ conditions, and for the binding incompatible trials by taking the mean value of the ‘full overlap-alternation’ and ‘no overlap-repetition’ conditions. Thus, we were able to compare trials in which an event-file binding needed to be reconfigured (i.e., binding incompatible) to trials in which an event-file binding did not need to be reconfigured (i.e., binding compatible) within the groups as well as between the groups (NT controls, AD(H)D). A mixed effects analysis of variance was used to statistically compare the conditions, including the within-subject factors “reconfiguration” (binding compatible, binding incompatible) and the between-subject factor “group” (NT, AD(H)D) for both accuracy (i.e., percentage of correct responses) and reaction time (in ms). Additionally, to control for whether age has a significant influence on the observed effects for task accuracy, the covariate “age” was added to the model, and a mixed-design ANCOVA was calculated. The significance level was set at p < 0.05 for the alpha threshold. All descriptive statistics will be reported by mean values (M) and standard error of mean (SE). In case of significant differences, Bonferroni-corrected post-hoc tests are reported.

Bayesian statistics were employed to assess the robustness of the significant behavioral findings. The Bayes Factor for *BF*_01_ was assessed and interpreted according to the Bayes Factor classification scheme (Wagenmakers et al., 2011): a BF value between 1 and 3 is considered anecdotal, between 3 and 10 as substantial, between 10 and 30 as strong, between 30 and 100 as very strong, and over 100 as extreme evidence for the null hypothesis (H_0_). The alternative hypothesis (H_1_) is supported anecdotally by values ranging from 1/3 to 1, substantially by values from 1/10 to 1/3, strongly by values from 1/30 to 1/10, extremely strongly by values from 1/100 to 1/30, and extremely by values below 1/100.

## 3 Results

### 3.1 Behavioral results

Regarding response accuracy, and independent of the group, participants responded significantly more accurately during binding compatible trials (91.0 % ± 0.8 %) than binding incompatible trials (76.5 % ± 1.3 %; main effect of reconfiguration: *F*(1,66) = 207.465, *p* < 0.001, *η_p_^2^* = 0.759, *BF*_01_ < 0.001). Hence, it can be concluded that the task modifications did not affect the event-file binding processes themselves and that the task works as a reliable measure adjusted for adolescents with and without AD(H)D, replicating earlier research showing that binding reconfiguration processes lead to a lower accuracy rate (e.g., Colzato, Warrens, et al., 2006; Graf et al., 2023, Kleimaker et al., 2020).

**Fig. 2:**
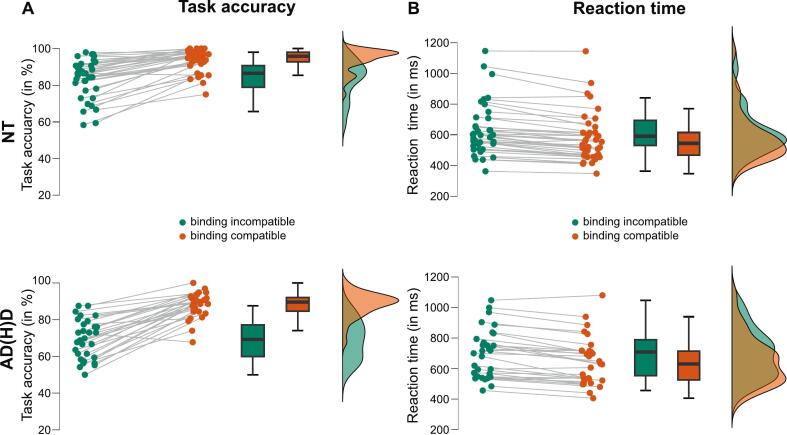
Behavioral results. A) Task accuracy. Raincloud plots and distribution of task accuracy are shown for both conditions (binding compatible, binding incompatible) for the NT controls (upper illustration) and the AD(H)D group (lower illustration) in percentage of correct responses. B) Reaction time. Raincloud plots and distribution of the reaction time for both conditions (binding compatible, binding incompatible) for the NT controls (upper illustration) and AD(H)D group (lower illustration) are displayed in milliseconds.

The data further show that AD(H)D participants (78.7 % ± 1.4 %) perform significantly worse in all conditions independently of the trial type compared to the NT control group (88.8 % ± 1.2 %; main effect of group: *F*(1,66) = 29.358, *p* < 0.001, *η_p_^2^* = 0.308, *BF*_01_ = 0.002). Importantly, there was a significant interaction effect “reconfiguration x group” (*F*(1,66) = 18.199, *p* < 0.001, *η_p_^2^* = 0.216, *BF*_01_ < 0.001). The significant interaction effect between “reconfiguration” and “group” reveals that the AD(H)D participants did not only perform worse in general in comparison to the NT controls, but that they also seem to have significantly more difficulties in reconfiguration processes for binding incompatible trials compared to trials in which no reconfiguration processes are demanded. Bonferroni-adjusted post-hoc tests showed that participants with AD(H)D performed significantly less accurately in binding compatible (*M_Diff_* = -0.058, *p* < 0.001, 95 %-CI[-0.089; −0.027]) and binding incompatible trials (*M_Diff_* = -0.144, *p* < 0.001, 95 %-CI[-0.196; −0.093]). Furthermore, while binding incompatible compared to binding compatible trials already led to a significantly higher percentage of incorrect responses in the NT control group (*M_Diff_* = 0.102, *p* < 0.001, 95 %-CI[0.075; 0.129]), the difference between the two conditions was even bigger for the AD(H)D group (*M_Diff_* = 0.188, *p* < 0.001, 95 %-CI[0.158; −0.218]). For visualization of the task accuracy results, please refer to Fig. 2A.

For reaction times, neither a significant main effect for “group” nor a significant interaction effect between “group” and “reconfiguration” was revealed (all *F’s* < 2.773, all *p’s* > 0.101, all *η_p_^2^* < 0.040). Yet, a significant main effect for “reconfiguration” was shown (*F*(1,66) = 114.289, *p* < 0.001, *η_p_^2^* = 0.634, *BF*_01_ < 0.001; see Fig. 2B). Specifically, all participants responded faster during compatible trials (612.469 ms ± 19.556 ms) compared to incompatible trials (664.267 ms ± 20.305 ms). Hence, reconfiguration processes seem to slow down participants’ response time, which is in line with earlier research on event-file binding processes (e.g., Graf et al., 2023).

#### 3.1.1 Age effects

Results of the mixed-design ANCOVA showed no evidence that the covariate “age” had a significant impact on the established main effects (“reconfiguration”, “group”) or their interaction (“reconfiguration x group”). After adjusting for age, the unadjusted significant results remained significant. Namely, all participants responded significantly more accurately during binding compatible trials (91.0 % ± 0.7 %) than binding incompatible trials (76.5 % ± 1.2 %; main effect of reconfiguration: *F*(1,65) = 11.566, *p* = 0.001, *η_p_^2^* = 0.151, *BF*_01_ = 0.031). Further, AD(H)D participants (78.9 % ± 1.3 %) performed significantly worse in all conditions independently of the trial type compared to the NT control group (88.6 % ± 1.1 %; main effect of group: *F*(1,65) = 31.90, *p* < 0.001, *η_p_^2^* = 0.329, *BF*_01_ < 0.001). Most importantly, a significant interaction effect “reconfiguration x group” (*F*(1,65) = 17.517, *p* < 0.001, *η_p_^2^* = 0.212, *BF*_01_ = 0.002) was revealed. Bonferroni-corrected post-hoc analyses revealed that, after adjusting for age, participants with AD(H)D performed significantly less accurately in binding compatible (*M_Diff_* = -0.054, *p* < 0.001, 95 %-CI[-0.083; −0.026]) and binding incompatible trials (*M_Diff_* = -0.138, *p* < 0.001, 95 %-CI[-0.187; −0.090]). Furthermore, while binding incompatible compared to binding compatible trials already led to a significantly higher percentage of incorrect responses in the NT control group (*M_Diff_* = 0.103, *p* < 0.001, 95 %-CI[0.077; 0.130]), the difference between the two conditions was even bigger for the AD(H)D group (*M_Diff_* = 0.187, *p* < 0.001, 95 %-CI[0.157; −0.218]), after adjusting for age.

### 3.2 Neurophysiological Data: Time-frequency analysis

**Fig. 3:**
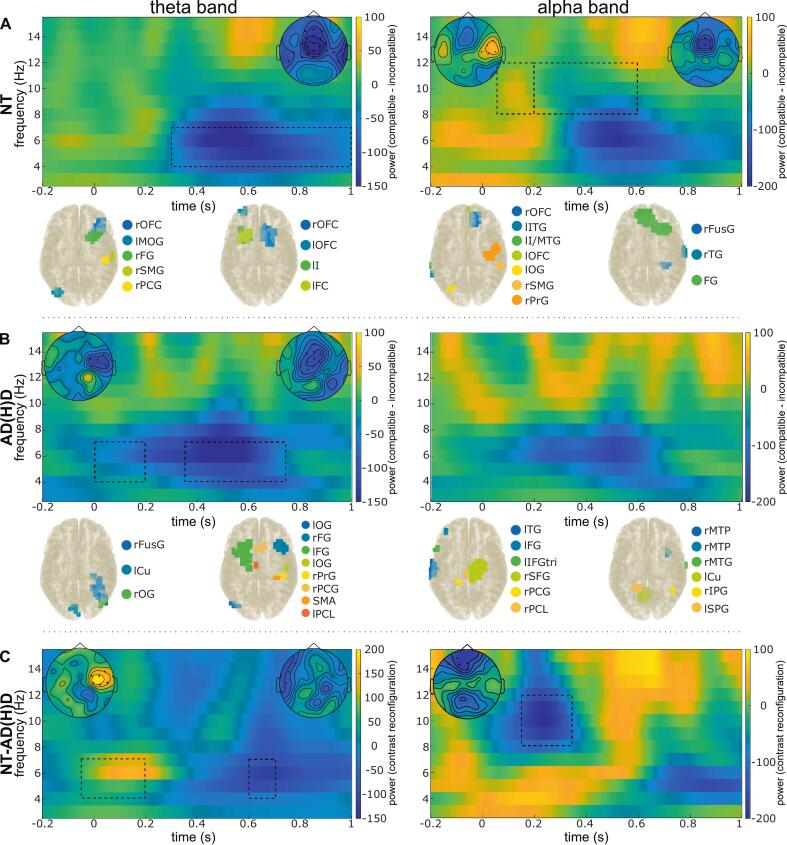
Time-frequency transformation within- and between-groups. A) and B) show time–frequency transformation, topoplots, and DBSCAN results of significant clusters for the reconfiguration effect (compatible–incompatible) within each group for alpha (right side) and theta band activity (left side). Time point zero reflects the presentation of the probe (S2). Within each frequency band, the power shown is averaged over the electrodes of the corresponding significant clusters as identified during cluster-based permutation testing. Plots depicting the DBSCAN clusters of reconstructed brain regions are displayed below the corresponding time–frequency transformation plots. Please refer to Table 2, Table 3 for a detailed description of all DBSCAN clusters and associated Brodmann areas. Time windows of DBSCANs are based on significant time–frequency clusters for each group, or from the other group, when no significant clusters were revealed. The time windows of each cluster are shown by boxes with dashed lines. C) shows time–frequency transformation and topoplots for the group difference (NT – AD(H)D) of the *reconfiguration contrast* (binding compatible – binding incompatible). The time windows of each cluster are shown by boxes with dashed lines.

Significant differences were revealed between compatible and incompatible conditions for both theta and alpha band activity via cluster-based permutation tests for within and between-group comparisons of NT controls and AD(H)D participants. Please refer to Fig. 3 for an overview of the time–frequency analysis results.

For theta band activity (TBA), significant negative differences (binding compatible < binding incompatible) were revealed for both groups, but within the NT control group from 300 – 1000 ms after stimulus onset (*t_sum_* = -97.271, *p_cluster_* < 0.001, at (fronto)central, parietal and temporal electrodes), and within the AD(H)D group from 0 – 200 ms (*t_sum_* = -26.994, *p_cluster_* = 0.014, at (fronto) central and (fronto)temporal electrodes) and further from 350 – 750 ms after stimulus onset (*t_sum_* = -26.994, *p_cluster_* = 0.016, at (fronto)central and parietal electrodes). Thus, both groups show a similar pattern of activity differences within the theta band, namely higher TBA during binding incompatible trials compared to binding compatible trials, even though this pattern seems to be more stable for a longer period within the NT control group compared to an earlier and more disjointed pattern within the AD(H)D group. By comparing the *reconfiguration contrast* (binding compatible – binding incompatible) between both groups, cluster-based permutation tests indicated a significant positive difference (NT controls > AD(H)D) in the theta band between approximately −50 and 200 ms (around the time of stimulus onset; *t_sum_* = 20.693, *p_cluster_* = 0.006, at prefrontal, (fronto)central and parietal electrodes), as well as a significant negative difference (NT controls < AD(H)D) around 600 to 700 ms after stimulus onset (*t_sum_* = -11.870, *p_cluster_* = 0.0490, at (fronto)temporal electrodes).

For alpha-band activity (ABA), cluster-based permutation tests only revealed significant differences for the NT control group, but not for the AD(H)D group. Specifically, a significant positive difference (binding compatible > binding incompatible) in ABA was revealed for the NT controls around 50 – 200 ms after stimulus onset (*t_sum_* = 12.523, *p_cluster_* = 0.038, at (fronto)central and (fronto)temporal electrodes), which then changed to a significant negative difference (binding compatible < binding incompatible) from 200 – 600 ms after stimulus onset (*t_sum_* = -17.181, *p_cluster_* = 0.012, at frontal electrodes). By comparing the *reconfiguration contrast* (binding compatible – binding incompatible) between both groups, cluster-based permutation tests indicated a significant negative difference (NT controls < AD(H)D) around 150 to 350 ms after stimulus onset (*t_sum_* = -17.397, *p_cluster_* = 0.024, at (pre)frontal electrodes).

### 3.3 Neurophysiological Data: DICS beamforming

After sensor-level differences between binding compatible and binding incompatible conditions were established for each group, the sources of these differences were reconstructed using DICS beamforming (Gross et al., 2001). Thereafter, they were clustered using the DBSCAN algorithm (Ester et al., 1996) and further reallocated based on the AAL atlas (Tzourio-Mazoyer et al., 2002). By that, local and anatomical relations better reflect the defined brain regions, and we obtain an estimation of which brain regions were likely activated during sensor-level differences for the contrast between binding incompatible and binding compatible trials in both groups. This was done for the time windows of significant sensor-level differences. In case no significant differences were found within a group, time windows were based on the time window of the group they are being compared to.

For TBA (−50 – 200 ms for both groups), five different clusters were revealed for the NT control group and two different clusters for the AD(H)D group. Clusters in the NT control group included right orbitofrontal and temporo-polar regions, left occipital cortex, right frontal areas, and right sensorimotor regions (postcentral, precentral, and supramarginal gyri). For the AD(H)D group, clusters included right fusiform, occipital, and temporal gyri, as well as left visual cortices. Later TBA was revealed to be associated with three different clusters for the NT control group (300 – 1000 ms), namely, right fronto-limbic and basal ganglia structures (including rectus gyrus, olfactory cortex, orbitofrontal cortex, insula, parahippocampal gyrus, amygdala, temporopolar area, and nucleus caudate, putamen, and pallidum), left frontal areas, and left insula. For the AD(H)D group (350 – 750 ms), four different clusters were revealed, including left occipital, bilateral frontal, left parietal, right sensorimotor (i.e., postcentral and precentral gyri), and bilateral supplementary motor areas. Please refer to Table 2 for an overview of all relevant clusters and corresponding Brodmann areas in the theta band.

**Table 2:** Results of DBSCAN clusters and associated Brodmann areas for averaged theta frequency band for each group (NT, AD(H)D).

Group	Time window	Clusters	Labels of figures	Neuroanatomical regions
NT	−50 – 200 ms	I	rOFC rTP	Right inferior and middle orbitofrontal cortex (BA 11) Right superior and middle temporopolar area (BA 38)
		II	lMOG	Left middle occipital gyrus (BA 19)
		III	rFG	Right superior and middle frontal gyrus (BA 8, 9, 10, 46)
		IV	rPCG rSMG	Right postcentral gyrus (BA 1, 2, 3) Right supramarginal gyrus (BA 40)
		V	rPCG rPrG	Right postcentral gyrus (BA 1, 2, 3) Right precentral gyrus (BA 4)
	300 – 1000 ms	I	rG.rectus rOC rOFC rI rPHG rAmyg rTP rCN, rPu, rPd	Right rectus gyrus (BA 11) Right olfactory cortex (BA 28) Right inferior, superior, and medial orbitofrontal cortex (BA 11) Right insula (BA 13, 14) Right parahippocampal gyrus (BA 28, 35, 36) Right amygdala (BA 27, 28) Right superior temporopolar area (BA 38) Right nucleus caudate, putamen, pallidum (structures within the basal ganglia, no BA associated)
		II	lOFC lFG lIFGop lIFGtri lPrG	Left middle and superior orbitofrontal cortex (BA 11) Left superior and middle frontal gyrus (BA 6, 9, 10, 46) Left inferior opercular frontal gyrus (Broca’s area, BA 44, 45) Left inferior triangular frontal gyrus (BA 45) Left precentral gyrus (BA 4)
		III	lI lFGtri	Left insula (BA 13, 14) Left inferior triangular frontal gyrus (BA 45)
AD(H)D	−50 – 200 ms	I	rFusG rOG rMTG	Right fusiform gyrus (BA 37) Right inferior and middle occipital gyrus (BA 19) Right middle temporal gyrus (BA 21)
		II	lCu lCS lSOG	Left cuneus (BA 17) Left calcarine sulcus (BA 17) Left superior occipital gyrus (BA 19)
	350 – 750 ms	I	lOG	Left inferior and middle occipital gyrus (BA 19)
		II	FG lPrG lPCL	Left and right superior and middle frontal gyrus (BA 8, 9, 10, 46, 47) Left precentral gyrus (BA 4) Left paracentral lobule (BA 5, 7)
		III	rPCG rPrG	Right postcentral gyrus (BA 1, 2, 3) Right precentral gyrus (BA 4)
		IV	SMA	Right and left supplementary motor area (BA 6)

Note. Neuroanatomical regions are grouped according to functional communalities and are based on Brodmann areas. Time windows of DBSCANs are based on significant time–frequency clusters for each group, or from the other group, when no significant clusters were revealed. Abbreviations: NT = neurotypical, AD(H)D = attention-deficit(−hyperactivity)-disorder.

For ABA (50 – 200 ms for both groups), five different clusters were revealed for the NT control group and three different clusters for the AD(H)D group. Clusters in the NT control group included right (orbito)frontal, left temporal, left occipital, and right parietal regions. For the AD(H)D group, clusters included left temporal, bilateral frontal, and right parietal regions. Later ABA (200 – 600 ms for both groups) was revealed to be associated with three different clusters for the NT control group, including right fusiform and right parahippocampal gyrus, right temporal regions, bilateral frontal regions, and right cingulum. For the AD(H)D group, three different clusters were revealed, including right temporal regions, left cuneus, bilateral precuneus, left calcarine sulcus, left cingulum, and parietal regions. Please refer to Table 3 for an overall overview of the relevant clusters and corresponding Brodmann areas in the alpha band.

**Table 3:** Results of DBSCAN clusters and associated Brodmann areas for averaged alpha frequency band for each group (NT, AD(H)D).

Group	Time window	Clusters		Labels of figures		Neuroanatomical regions		
NT	50 – 200 ms	I		rOFC rG.rectus		Right superior and middle orbitofrontal cortex (BA 11) Right rectus gyrus (BA 11)		
		II		lTG		Left inferior and middle temporal gyrus (BA 21)		
		III		lOG		Left superior and middle occipital gyrus (BA 19)		
		IV		rSMG rIPG		Right supramarginal gyrus (BA 40) Right inferior parietal gyrus (BA 39, 40)		
		V		rPrG rPCG rMFG		Right precentral gyrus (BA 4) Right postcentral gyrus (BA 1, 2, 3) Right middle frontal gyrus (BA 6)		
	200 – 600 ms	I		rFusG rPHG		Right fusiform gyrus (BA 37) Right parahippocampal gyrus (BA 28, 35, 36)		
		II		rTG		Right superior and middle temporal gyrus (BA 21)		
		III		SFGmed FG rACC, rMCC		Right and left superior medial frontal gyrus (BA 8) Right and left superior and middle frontal gyrus (BA 8, 9, 10, 46) Right anterior and middle cingulum (BA 24, 31, 32)		
AD(H)D	50 – 200 ms	I		lTG		Left superior, middle, and inferior temporal gyrus (BA 21)		
		II		lMFG lIFGtri		Left middle frontal gyrus (BA 46) Left inferior triangular frontal gyrus (BA 45)		
		III		rPrG rPCG rSFG rPCL rSMA		Right precentral gyrus (BA 4) Right postcentral gyrus (BA 1, 2, 3) Right superior frontal gyrus (BA 8) Right paracentral lobule (BA 4) Right supplementary motor area (BA 6)		
	200–––600 ms	I		rMTP rMTG		Right middle temporopolar area (BA 38) Right middle temporal gyrus (BA 21)		
		II		lCu PCu lCS lPC		Left cuneus (BA 17) Left and right precuneus (BA 7) Left calcarine sulcus (BA 17) Left posterior cingulum (BA 23, 31)		
		III		lSPG lPCu lPCG		Left superior parietal gyrus (BA 5, 7) Left precuneus (BA 7) Left postcentral gyrus (BA 1, 2, 3)		

Note. Neuroanatomical regions are grouped according to functional communalities and are based on Brodmann areas. Time windows of DBSCANs are based on significant time–frequency clusters for each group, or from the other group, when no significant clusters were revealed. Abbreviations: NT = neurotypical, AD(H)D = attention-deficit(−hyperactivity)-disorder.

## 4 Discussion

Deficits in cognitive control and goal-directed behavior have frequently been linked to AD(H)D. Yet, the integration of perception and action – an essential aspect of cognition and central to goal-directed behavior – and its underlying neurophysiological mechanisms have received little attention in AD(H)D research. Hence, the objective of this study was to investigate perception–action integration processes and the neurophysiological mechanisms involved in adolescents with AD(H)D compared to NT controls. Overall, our study results suggest that individuals with AD(H)D experience greater difficulties in reconfiguring and updating existing perception–action representations. This was evident not only in the poorer general behavioral performance but especially in the performance in binding incompatible trials (i.e., trials requiring reconfiguration processes). Neurophysiological results stress the importance of modulating both TBA and ABA for the successful management of perception–action representations and further support recent evidence suggesting that AD(H)D might reflect a disorder of deficient alpha band modulation (Gholamipourbarogh et al., 2025, Graf et al., 2024, Ippolito et al., 2022, Lenartowicz et al., 2014, Lenartowicz et al., 2019, Michelini et al., 2022).

Behavioral results of the study replicated previous findings showing that participants generally perform less successfully (i.e., less accurately and more slowly) in binding incompatible conditions compared to binding compatible ones since these conditions prompt an update or reconfiguration of a pre-existing stimulus–response association (Colzato et al., 2006a, Colzato et al., 2006b, Dilcher et al., 2021, Eggert et al., 2021, Graf et al., 2023, Hommel, 2004, Takacs et al., 2020a, Takacs et al., 2020b). This pattern was observed independently of the group they belong to. Furthermore, adolescents with AD(H)D generally performed worse than neurotypical controls, which is consistent with prior research suggesting that patients with AD(H)D show deficits in cognitive control tasks (e.g., Ahmadi et al., 2014, Castellanos and Tannock, 2002, Randall et al., 2009). Most importantly, patients with AD(H)D also exhibited stronger binding effects − that is, they were even more affected by (un)binding and reconfiguration processes of earlier established perception–action presentations. Behaviorally, this was indicated by the AD(H)D group performing not only worse in general but also specifically in binding incompatible trials when previously established perception–action links needed to be updated. This suggests that AD(H)D-associated difficulties in the efficient reconfiguration of perception–action representations are not limited to the context of response inhibition (Graf et al., 2024) but expand across contexts (such as response selection). By that, these findings provide novel insights into how cognitive control and executive functioning deficits may arise in AD(H)D and support existing theories suggesting executive dysfunction to be one of the core components that underlie the complex neuropsychological profile of AD(H)D, and therefore its symptomatology development (e.g., Willcutt et al., 2005).

Neurophysiological data suggest that distinct patterns of modulated oscillatory activity in response to varying task demands may underlie the observed behavioral deficits in the AD(H)D group compared to NT controls. Both groups exhibited increased TBA during binding incompatible compared to binding compatible trials. However, this increase appears to be more sustained and temporally stable in the NT control group when compared to an earlier occurring and more disjointed pattern within the AD(H)D group. This enhanced TBA during binding incompatible trials aligns with previous research suggesting that especially frontal TBA is increased when confronted with higher cognitive demands (Graf et al., 2024, Prochnow et al., 2021, Prochnow et al., 2022, Rawish et al., 2024). This is thought to reflect the increased need to monitor cognitive processes and to update earlier established mental representations (Beste et al., 2023, Prochnow et al., 2021, 2022). Evaluating the involved sources likely underlying TBA for the *reconfiguration contrast* (binding compatible – binding incompatible) within both groups further substantiates the involved cognitive processes. Activation of regions of the ventral stream − such as the fusiform and parahippocampal gyrus – along with the temporal pole and occipital cortices, has been associated with the flexible management of event-files (Eggert et al., 2023, Kühn et al., 2011) and further reflects the visual processing of the stimuli of the task (i.e., identification and categorization of visual stimuli; Dehaene et al., 2002, Devlin et al., 2006, Goodale et al., 2005, Grill-Spector and Weiner, 2014). Notably, next to the reported frontal activity within both groups, orbitofrontal regions − areas previously shown to be involved in receiving memory information linked to the retrieval of previously established representations (Eggert et al., 2022) and in adaptation processes of goal-directed behavior (Rolls, 2004, Rudebeck and Rich, 2018) − were shown to be activated only within the NT control group*.* All of this underlines TBA’s important role during the management of perception–action representations and leads to the suggestion that TBA modulations in response to heightened cognitive demands are not deficient per se in AD(H)D adolescents. Yet, based on the literature discussed, adolescents with AD(H)D possibly lack (higher-frequency) modulatory mechanisms to guide these TBA modulations efficiently.

Indeed, the study further shows that successful management of perception–action representations does not depend on TBA modulation alone, but also significantly on ABA and its potential role in controlling these theta-band processes via bottom-up attentional and top-down control (e.g., Beste et al., 2023, Graf et al., 2024). Strikingly, the AD(H)D group showed a reduced ability to modulate their ABA when updating existing perception–action representations, with no significant ABA-modulated differences across task demands. This stands in contrast to their neurotypical peers, who show a specific ABA modulated pattern: just after stimulus onset, ABA was more strongly modulated in binding compatible compared to incompatible conditions, which changed to a stronger modulation of binding incompatible trials compared to compatible ones just before the motor response. Heightened (fronto)central and (fronto)temporal ABA during retrieval in compatible trials might reflect a way to process incoming information more efficiently and to increase attentional control. This, in turn, may help to ignore or disregard trial-irrelevant information, allowing for more efficient retrieval of previously formed stimulus–response associations (Bonnefond and Jensen, 2012, Foxe and Snyder, 2011, Jamous et al., 2024, Janssens et al., 2018, Klimesch et al., 2007, Prochnow et al., 2022). In contrast, enhanced (pre)frontal ABA during reconfiguration processes of binding incompatible trials, especially around the time of response, may reflect the greater cognitive demands associated with reconfiguring existing stimulus–response links. This aligns with prior research suggesting that ABA is enhanced under heightened attentional and working memory demands (Janssens et al., 2018, Jensen et al., 2002, Lenartowicz et al., 2018). Crucially, this dynamic modulatory pattern of ABA was absent within the AD(H)D group, suggesting that this group possibly lacks the modulating effects of ABA on theta-band processes in response to the need to update existing perception–action representations. This is in line with prior research suggesting AD(H)D to be characterized by deficient alpha band modulation (Graf et al., 2024, Ippolito et al., 2022, Lenartowicz et al., 2014, Lenartowicz et al., 2019, Michelini et al., 2022). Furthermore, while the study results suggest that similar brain regions were likely activated in both groups when evaluating the sources underlying ABA for the *reconfiguration contrast*, some differences for ABA become apparent too. Particularly, both groups showed activation in frontal, temporal, and parietal sensorimotor regions shortly after stimulus onset, likely reflecting sensorimotor integration of the presented stimulus (Borra et al., 2017, Elsner et al., 2002, Kühn et al., 2011, Van Essen et al., 2016). However, early occipital ABA modulation, which was previously shown to be related to retrieval processes of existing stimulus–response associations and also to play a role in serving as a top-down control over TBA-related processes and improved attentional control for filtering (ir)relevant information (Beste et al., 2023, Janssens et al., 2018, Prochnow et al., 2022), seemed to be only activated for the NT controls. Moreover, while the NT control group mainly engaged networks associated with memory and cognitive control processes (including fusiform gyrus, parahippocampal gyri, frontal cortices, and cingulum) just before and during the response period, the AD(H)D group showed greater activation in visual-spatial and sensory processing areas (including precuneus, cuneus, calcarine sulcus, and parietal regions). This further highlights alpha-band related differences linked to possibly partly differently activated brain regions.

Alpha band modulation in adolescents with AD(H)D in response to varying task demands may not be as developed yet as in NT controls due to the interplay of several factors. First, the maturation of fast-wave brain activity, such as alpha oscillations, has generally been shown to develop later compared to slower brain rhythms like TBA (Cellier et al., 2021, Gasser et al., 1988). Additionally, developmental delays in brain maturation observed in AD(H)D (Shaw et al., 2007) may further slowdown the developmental trajectories of neural oscillations and brain network integration. Moreover, consistent with previous research on prefrontal dysfunction in AD(H)D (Arnsten, 2009, Bush, 2011, Silk et al., 2008), the current findings suggest that alpha-band related top-down processes − particularly in prefrontal regions − supports perception–action integration in NT controls, but appears reduced or absent in the AD(H)D group. This lack of top-down regulation might be linked to known alterations in prefrontal cortex functioning in AD(H)D patients and may explain the group’s diminished alpha band responsiveness to task demands. Instead, the current study results suggests that adolescents with AD(H)D may attempt to compensate for their deficient alpha band modulation by relying more heavily and earlier on increased efforts to modulate TBA just after stimulus onset. By that, the current study emphasizes the critical role of modulating both ABA and TBA efficiently for successfully managing perception–action integration processes and demonstrates that modulating TBA alone is not sufficient (Beste et al., 2023). Future research with adult AD(H)D participants should investigate whether alpha-band modulation improves with brain maturation and whether it becomes more efficient in updating perception–action representations. This is particularly relevant since earlier research showed that the management of stimulus–response associations generally works more efficiently when approaching adulthood (Dilcher et al., 2021).

There are limitations to the study. Although efforts were made to adjust and simplify the task for adolescents with AD(H)D, the large number of participant exclusions should be considered when drawing conclusions from the study results. Possibly, we studied a rather high-functioning or less severely affected AD(H)D sample. Future studies might benefit from adjusting the task to be more naturalistic or relevant for the studied sample. Further, while we tried to minimize the effects of AD(H)D medication on study performance, we cannot rule out that they contributed to the obtained effects. Future studies might benefit from stricter inclusion criteria regarding medication use to better isolate neurophysiological differences. Additionally, while previous literature expected the effect of gender on cognitive development to be quite minimal (e.g., Grissom and Reyes, 2019, Hyde, 2016), we cannot rule out for certain that gender did not contribute to the observed effects. Lastly, individual brain scans could not be performed, limiting the reliability of the specific Brodmann areas mapped from the source reconstruction. Brain area localization results should thus be interpreted with caution.

In summary, the study provides novel insights into how cognitive control and executive functioning deficits may arise in adolescents with AD(H)D by being among the first to investigate the underlying neurophysiological mechanisms of perception–action integration in the context of response selection in AD(H)D. Next to general deficits, study results suggest that adolescents with AD(H)D show specific impairments when being confronted with conditions in which previously established perception–action representations do not remain intact and need to be reconfigured. Findings of the study further indicate that this likely develops due to a reduced ability to modulate ABA in response to heightened cognitive demands, which was suggested to exert crucial bottom-up attentional and top-down control processes over TBA. By that, the study highlights the importance of modulating both TBA and ABA for a successful reconfiguration of perception–action representations.

## Funding sources

This work was supported by a Grant from the Deutsche Forschungsgemeinschaft (DFG) FOR 2698 and by the Federal Ministry of Education and Research (Bundesministerium für Bildung und Forschung, BMBF) as part of the German Center for Child and Adolescent Health (DZKJ) under the funding code 01GL2405B.

## CRediT authorship contribution statement

**Katharina Graf:** Writing – review & editing, Writing – original draft, Visualization, Investigation, Formal analysis, Data curation, Conceptualization. **Roula Jamous:** Writing – review & editing, Writing – original draft, Methodology, Investigation, Conceptualization. **Annet Bluschke:** Writing – review & editing, Writing – original draft, Supervision, Conceptualization. **Christian Beste:** Writing – review & editing, Writing – original draft, Validation, Supervision, Resources, Project administration, Methodology, Conceptualization.

## Declaration of competing interest

The authors declare that they have no known competing financial interests or personal relationships that could have appeared to influence the work reported in this paper.

## Data Availability

The data is available in OSF
